# Establishment of a National Surveillance and Management System for *Candida auris* Infection in the Republic of Korea

**DOI:** 10.56786/PHWR.2026.19.38.2

**Published:** 2026-09-01

**Authors:** Nam Yi Kim, Su-Yeon Lee, Seung Gul Lee, Jae Woo Park

**Affiliations:** Division of Healthcare Associated Infection Control, Department of Healthcare Safety and Immunization, Korea Disease Control and Prevention Agency Cheongju Korea

**Keywords:** *Candida auris*, Cross infection, Sentinel surveillance, Infection control

## Abstract

**Objectives:**

*Candida auris* is an emerging fungal pathogen that requires national preparedness because of its antifungal resistance, environmental persistence, healthcare-associated transmission, and outbreak potential. Following the detection of domestic clade I cases in the Republic of Korea (ROK) and healthcare-associated transmission, the Korea Disease Control and Prevention Agency established a national surveillance and management framework. This report describes the designation of *C. auris* infection as a Class 4 notifiable infectious disease and a healthcare-associated infectious disease and the development of this framework.

**Methods:**

International and domestic epidemiological evidence, including domestic clade I cases and a 2025 survey of 155 healthcare facilities, was reviewed alongside guidance from the World Health Organization, the US Centers for Disease Control and Prevention, and the European Centre for Disease Prevention and Control. Expert consultations and committee deliberations informed reporting criteria and the development of surveillance and management guidelines, treatment recommendations, and supporting measures.

**Results:**

*C. auris* infection was designated as a Class 4 notifiable infectious disease and a healthcare-associated infectious disease. Since March 29, 2026, cases diagnosed at 368 sentinel surveillance institutions nationwide must be reported within 7 days of diagnosis. National management guidelines and treatment recommendations were issued, health insurance coverage for isolation-room inpatient fees was introduced, and surveillance personnel received training. A complementary surveillance system was established to collect antifungal susceptibility and molecular epidemiological data.

**Conclusions:**

These measures established a national framework for surveillance and management encompassing mandatory reporting, infection prevention and control, treatment recommendations, health insurance coverage, and complementary surveillance. Continued integration of sentinel and complementary surveillance will strengthen the evidence base on *C. auris* epidemiology and transmission in the ROK and inform further improvements in national surveillance, prevention, and control policies.

**Key messages**

**① What is known previously?**

*Candida auris* is an emerging fungal pathogen characterized by multidrug resistance, environmental persistence, healthcare-associated transmission, and outbreak potential. The World Health Organization has designated *C. auris* as a critical priority fungal pathogen.

**② What new information is presented?**

The Korea Disease Control and Prevention Agency designated *C. auris* infection as a Class 4 notifiable infectious disease and healthcare-associated infectious disease. It established a national surveillance and management framework encompassing mandatory reporting, infection prevention and control, treatment recommendations, health insurance coverage, and complementary surveillance.

**③ What are implications?**

The national surveillance and management framework is expected to strengthen the evidence base on *C. auris* epidemiology and transmission in the Republic of Korea and inform further improvements in national surveillance, prevention, and control policies.

## Introduction

*Candida auris* is an emerging fungal pathogen that has been increasingly reported in healthcare facilities worldwide since it was first identified in Japan in 2009. *C. auris* exhibits high rates of resistance to multiple antifungal agents, including fluconazole, and some strains are also resistant to amphotericin B and echinocandins [1]. Its prolonged persistence in healthcare environments and capacity for patient-to-patient transmission can lead to outbreaks in healthcare facilities. Because conventional biochemical identification methods may misidentify *C. auris* as other Candida species, rapid diagnosis and infection control are increasingly important. In view of its high rates of antifungal resistance, potential for sustained transmission and outbreaks in healthcare facilities, and difficulty of identification and eradication from healthcare environments, the World Health Organization (WHO) classified *C. auris* as a critical-priority pathogen in its 2022 Fungal Priority Pathogens List [2]. The US Centers for Disease Control and Prevention (CDC) and the European Centre for Disease Prevention and Control (ECDC) also recommend early detection in healthcare facilities, patient isolation, contact precautions, environmental control, and continuous surveillance [3,4]. These recommendations underscore the need for surveillance systems that enable early interruption of transmission in healthcare facilities and continuous monitoring of national epidemiological trends. In the Republic of Korea (ROK), after a clade I strain was identified in a blood specimen in 2018, additional clade I cases were reported [5]. In 2022, healthcare-associated outbreaks and transmission of clade I *C. auris* linked to a patient transferred from abroad were confirmed [5,6]. These events highlighted the need for a national surveillance and management system to prepare for and respond to sustained transmission in healthcare facilities. Following analyses of the epidemiological situation and infection control practices in healthcare facilities [6,7], expert consultations, and deliberations by relevant committees, *C. auris* infection was designated as a Class 4 notifiable infectious disease and a healthcare-associated infectious disease. Because the number of *C. auris* cases in the ROK remains limited and its epidemiological characteristics have not been fully characterized, a surveillance system was needed that could promptly use the existing healthcare-associated infectious disease sentinel surveillance network while rapidly capturing national epidemiological data. Accordingly, a phased strategy was adopted first, to establish a Class 4 notifiable infectious disease management system based on the nationwide network of healthcare-associated infectious disease sentinel surveillance institutions and subsequently, to refine the scope of surveillance and reporting criteria as epidemiological data accumulate. This report describes the designation of *C. auris* infection as a Class 4 notifiable infectious disease and the establishment of a national surveillance and management system, including reporting, infection prevention and control, treatment support, and complementary surveillance.

## Methods

### 1. Review of Domestic and International Epidemiological Trends and Management Systems

To establish a national management system for *C. auris* infection, we reviewed domestic and international epidemiological trends and management approaches. The domestic review covered reported *C. auris* cases, the epidemiology of clade I, healthcare-associated outbreaks, infection control practices, and the operation of management systems in healthcare facilities. The international review focused on guidelines and recommendations published by the WHO, CDC, and ECDC and compared notifiable disease surveillance systems and infection control policies in healthcare facilities across selected countries [2-4].

### 2. Survey of Healthcare Facilities in the Republic of Korea

To assess the occurrence of *C. auris* and infection control practices in Korean healthcare facilities, a nationwide survey of 155 healthcare facilities was conducted in 2025. The survey examined facility-level occurrence, testing practices, patient management and isolation measures, environmental management, and the operation of infection prevention and control programs. The findings provided baseline data for establishing a national surveillance and management system and developing reporting criteria [7].

### 3. Designation as a Class 4 Notifiable Infectious Disease and Establishment of Reporting Criteria

Based on domestic and international epidemiological evidence and the survey findings, we assessed whether *C. auris* infection should be designated as a Class 4 notifiable infectious disease and reviewed the reportable case categories, reporting criteria, reporting timeframe, and operational plan. Beginning in December 2024, the process included expert consultations, reviews by relevant academic societies, public hearings, and sequential deliberations by the Healthcare-associated Infection Expert Committee and the Infectious Disease Management Committee.

### 4. Review of Plans to Establish a National Surveillance and Management System

Given the limited data on the number of cases and epidemiological characteristics in the ROK, we evaluated the feasibility of using the existing healthcare-associated infectious disease sentinel surveillance system for case reporting. We also developed management guidelines, treatment recommendations, personnel training, and support measures for isolation care to facilitate implementation.

### 5. Development of Plans to Promote Complementary Surveillance

To address the limitations of epidemiological data collected through sentinel surveillance, we developed a complementary surveillance plan linking antifungal susceptibility testing and molecular epidemiological analysis of isolates obtained from healthcare facilities.

## Results

### 1. Designation as a Class 4 Notifiable Infectious Disease and Establishment of Reporting Criteria

A comprehensive review of domestic epidemiological data, international management approaches, and expert opinions indicated that *C. auris* infection should be managed through a system capable of immediately using the existing healthcare-associated infectious disease sentinel surveillance network while rapidly accumulating epidemiological data in the ROK. Accordingly, the “Kinds of Infectious Diseases Designated by the Commissioner of the Korea Disease Control and Prevention Agency” and the “Diagnostic Criteria for Infectious Disease Reporting” were revised to designate *C. auris* infection as a Class 4 notifiable infectious disease and a healthcare-associated infectious disease and to define reportable case categories and diagnostic criteria. Since March 29, 2026, cases diagnosed at healthcare-associated infectious disease sentinel surveillance institutions nationwide must be reported within 7 days of diagnosis (Table 1).

### 2. Establishment of a National Surveillance System

*C. auris* infection was incorporated into the existing healthcare-associated infectious disease sentinel surveillance system, which comprises a nationwide network of 368 sentinel surveillance institutions. The system continuously collects data on case numbers, patient characteristics, distribution by healthcare facility type, and regional occurrence, enabling systematic analysis of epidemiological patterns and transmission characteristics in the ROK.

### 3. Establishment of Infection Control and Treatment Support Systems

In conjunction with the designation of *C. auris* infection as a notifiable infectious disease, the “2026 Guidelines for the Management of *Candida auris* Infection” and the “Recommendations for the Treatment of *Candida auris* Infection” were developed and distributed to healthcare facilities nationwide [8,9]. The management guidelines set out infection control principles for preventing transmission in healthcare facilities, including early case detection and reporting, isolation, contact precautions, and environmental disinfection; the key recommendations are summarized in Table 2 [8]. In addition, health insurance coverage for isolation-room inpatient fees was introduced to reduce the financial burden of isolation care and support implementation of the recommended infection control measures [10].

### 4. Training for Personnel and Support for System Implementation

Before implementation, personnel at regional disease response centers, local governments, and healthcare-associated infectious disease sentinel surveillance institutions nationwide received training on reporting criteria and procedures, infection control principles, and epidemiological investigation methods. The training covered policy changes arising from the notifiable disease designation and standardized reporting and infection control procedures across institutions, thereby supporting stable implementation of the system.

### 5. Establishment of a Complementary Surveillance System

To supplement the epidemiological data collected through sentinel surveillance, a complementary surveillance system was established to collect *C. auris* isolates from small- and medium-sized hospitals and long-term care hospitals for antifungal susceptibility testing and molecular epidemiological analysis. This system provides a basis for continuous assessment of circulating clades and antifungal resistance patterns in the ROK and for detailed analysis of epidemiological and transmission patterns through linkage with clinical and epidemiological data from sentinel surveillance. The national management system integrates infection control, treatment support, and complementary surveillance around mandatory reporting by sentinel surveillance institutions (Table 3).

## Discussion

*C. auris* is an emerging fungal pathogen of growing global public health importance because of its antifungal resistance, sustained transmission in healthcare facilities, and outbreak potential. The WHO classified *C. auris* as a critical-priority pathogen in its 2022 Fungal Priority Pathogens List [2], and the United States and European countries have strengthened healthcare-based surveillance and infection control strategies [3,4]. In the ROK, clade I cases [5] and healthcare-associated outbreaks and transmission [5,6] have been confirmed since 2018, demonstrating the need for systematic surveillance and management at the national level.

However, limited data on the number of cases and their epidemiological characteristics in the ROK constrained the immediate implementation of comprehensive surveillance. Therefore, a Class 4 notifiable infectious disease management system was first established using the existing healthcare-associated infectious disease sentinel surveillance network to rapidly generate domestic epidemiological data. Importantly, the initiative extended beyond notifiable disease designation to establish an integrated national system encompassing reporting, infection control, treatment support, and complementary surveillance.

Use of the existing healthcare-associated infectious disease sentinel surveillance system enabled rapid initiation of nationwide surveillance while minimizing the time and administrative burden of establishing a new system. In addition, the development of management guidelines and treatment recommendations [8,9], together with health insurance coverage for isolation-room inpatient fees, provided financial support for healthcare facilities to implement the recommended infection control measures more consistently [10]. This strategy provides an important policy foundation for patient isolation and prevention of transmission in healthcare facilities while reducing the associated financial burden.

Sentinel surveillance is effective for monitoring nationwide epidemiological trends but cannot capture every case. Complementary surveillance will therefore include antifungal susceptibility testing and molecular epidemiological analysis of isolates from small- and medium-sized hospitals and long-term care hospitals. The distribution of circulating clades and transmission characteristics in the ROK will be assessed continuously in conjunction with clinical and epidemiological data. These data will support more accurate characterization of circulation patterns and future reassessment of the surveillance system and reporting criteria. In conclusion, designation of *C. auris* infection as a Class 4 notifiable infectious disease represents the first step toward a national management system that prevents transmission in healthcare facilities while rapidly accumulating epidemiological data. Continued linkage of sentinel and complementary surveillance, together with antifungal susceptibility and molecular epidemiological data, will support further development of a surveillance and management system tailored to the epidemiological situation in the ROK.

## Figures and Tables

**Table 1:** Reporting criteria for *Candida auris* infection

Category	Reporting criteria
Cases subject to reporting	Patients and carriers
Patient	A person from whose blood *Candida auris* is isolated
Carrier	A person from whose clinical specimen other than blood *C. auris* is isolated, excluding ear specimens
Reporting period	Cases diagnosed at sentinel surveillance institutions must be reported within 7 days of diagnosis
Clinical manifestations	Clinical manifestations of systemic infection symptoms, such as sepsis, soft tissue infection, and surgical site infection; persistent signs or symptoms of infection that do not respond to antifungal therapy
Laboratory criteria for diagnosis	Isolation and identification of *C. auris* from a clinical specimen, such as blood or urine, excluding ear specimens

**Table 2:** Key recommendations for infection prevention and control of *Candida auris* in healthcare facilities

Category	Key recommendations
Contact precautions	Apply standard and contact precautions. Perform hand hygiene before and after contact with a patient, carrier, or the surrounding environment, and wear gloves and a gown.
Patient isolation	Place patients in a single room whenever possible. If a single room is unavailable, cohort patients infected or colonized with the same organism or maintain a distance of ≥1 m between beds and use a physical barrier. Maintain isolation throughout hospitalization.
Equipment and environmental cleaning	Use dedicated or disposable medical equipment. Thoroughly clean and disinfect shared equipment and high-touch environmental surfaces, with terminal cleaning after patient discharge.
Screening and contact management	Consider screening patients with a history of infection or colonization or an epidemiologic link to a case. Using a single swab, collect a composite specimen from both axillae and groins.
Movement and transfer	Limit unnecessary patient movement. During intrafacility movement or interfacility transfer, communicate the patient’s infection or colonization status and the necessary infection prevention and control measures.

**Table 3:** National surveillance and management framework for *Candida auris* infection

Reporting system	Infection prevention and control	Treatment support	Complementary surveillance
Reporting by 368 sentinel surveillance institutions within 7 days of diagnosis	Early detection and isolation, contact precautions, and environmental disinfection	Treatment recommendations and health insurance coverage for isolation-room inpatient fees	Collection of *C. auris* isolates, antifungal susceptibility testing, and molecular epidemiological analysis
