# Supplementary material for: Establishment of a National Surveillance and Management System for Candida auris Infection in the Republic of Korea
Source: Public Health Wkly Rep. 2026 Sep 1;19(38):1620–32. doi: 10.56786/PHWR.2026.19.38.2 (PMC13655513; doi:10.56786/PHWR.2026.19.38.2)
Supplement: Translation

# Translation: 칸디다 오리스 감염증 국가 감시ㆍ관리체계 구축

**Authors:** 남이 김, 수연 이, 승걸 이, 재우 박

**Affiliations:** 질병관리청 의료안전예방국 의료감염관리과

**Keywords:** 칸디다 오리스, 의료관련감염, 표본감시, 감염예방관리

## Abstract

**목적:**

칸디다 오리스(*Candida auris*)는 항진균제 내성, 의료환경에서의 장기 생존, 의료기관 내 전파 및 집단발생 가능성으로 국가 차원의 감시와 관리가 필요한 신종 진균 병원체이다. 국내에서도 칸디다 오리스 Clade I 발생과 의료기관 내 집단발생이 확인됨에 따라 국가 감시ㆍ관리체계를 구축하였다. 본 보고서는 칸디다 오리스 감염증의 제4급 법정감염병 지정과 국가 관리체계 구축 과정을 소개하고자 하였다.

**방법:**

국내외 발생 현황과 관리 동향, 세계보건기구, 질병통제예방센터 및 유럽질병예방통제센터의 권고사항, 국내 Clade I 발생 사례 및 2025년 국내 의료기관 155개소 실태조사 결과를 검토하였다. 이를 바탕으로 전문가 자문과 관련 위원회 심의를 거쳐 신고기준을 마련하고 감시체계, 관리지침, 치료 권고안 및 지원체계를 구축하였다.

**결과:**

칸디다 오리스 감염증을 제4급 법정감염병 및 의료관련감염병으로 지정하고, 2026년 3월 29일부터 전국 368개 의료관련감염병 표본감시기관을 대상으로 진단 후 7일 이내 신고체계를 운영하였다. 관리지침과 치료 권고안을 마련하여 배포하였으며, 격리실 입원료에 건강보험 급여를 적용하고 담당자 교육을 실시하였다. 또한 표본감시와 연계한 보완적 감시체계를 구축하여 항진균제 감수성 및 분자역학 자료를 지속적으로 수집할 기반을 마련하였다.

**결론:**

칸디다 오리스 감염증의 제4급 법정감염병 지정으로 신고체계, 감염관리, 치료지원 및 보완적 감시체계를 포함한 국가 관리체계가 구축되었다. 향후 표본감시와 보완적 감시를 연계하여 국내 역학정보와 항진균제 내성 및 분자역학 자료를 지속적으로 축적함으로써 국내 상황에 적합한 감시 및 관리체계를 발전시켜 나갈 예정이다.

**핵심요약**

**① 이전에 알려진 내용은?**

칸디다 오리스는 항진균제 내성, 환경 생존력, 의료기관 내 전파 및 집단발생 위험으로 인해 세계보건기구가 최우선 대응이 필요한 진균 병원체로 지정한 신종 병원체이다.

**② 새로이 알게 된 내용은?**

국내 역학자료를 신속히 확보하고 기존 의료관련감염병 표본감시체계를 활용하기 위하여 칸디다 오리스 감염증을 제4급 법정감염병 및 의료관련감염병으로 지정하고, 신고체계, 감염관리, 치료 권고안과 건강보험 지원 및 보완적 감시체계를 포함한 국가 관리체계를 구축하였다.

**③ 시사점은?**

법정감염병 지정과 표본감시체계 운영은 국내 발생 양상과 전파 특성에 대한 근거를 지속적으로 축적하고 향후 감시체계와 관리정책을 고도화하기 위한 기반이 될 것으로 기대된다.

## 서 론

칸디다 오리스(*Candida auris*)는 2009년 일본에서 처음 보고된 이후 전 세계 의료기관에서 발생이 지속적으로 증가하고 있는 신종 진균 병원체이다. 칸디다 오리스는 fluconazole을 비롯한 여러 항진균제에 대한 높은 내성을 보이며, 일부 균주는 amphotericin B 및 echinocandin에도 내성을 나타내는 다제내성 특성을 가지고 있다[1]. 또한 의료환경에서 장기간 생존하고 환자 간 전파가 가능하여 의료기관 내 집단발생을 유발할 수 있으며, 기존 생화학적 동정방법으로는 다른 *Candida* 종으로 오인될 가능성이 있어 신속한 진단과 감염관리의 중요성이 지속적으로 강조되고 있다. 세계보건기구(World Health Organization, WHO)는 높은 항진균제 내성률, 의료기관 내 지속적인 전파와 집단발생 가능성, 동정 및 의료환경에서의 완전한 제거가 어려운 특성을 근거로 2022년 칸디다 오리스를 Fungal Priority Pathogens List의 최우선 대응군(Critical Priority Group)으로 지정하였다[2]. 미국 질병통제예방센터(Centers for Disease Control and Prevention, CDC)와 유럽질병예방통제센터(European Centre for Disease Prevention and Control, ECDC) 역시 의료기관 내 조기 발견, 환자 격리, 접촉주의, 환경관리 및 지속적인 감시체계 운영을 권고하고 있다[3,4]. 이러한 국제적 권고는 의료기관 내 전파를 조기에 차단하고 국가 차원의 발생 현황을 지속적으로 파악하기 위한 감시체계 구축의 필요성을 뒷받침하고 있다. 국내에서는 2018년 혈액 검체에서 칸디다 오리스 Clade I 균주가 확인된 이후 국내 Clade I 발생이 보고되었고[5], 2022년에는 의료기관 내 Clade I 집단발생과 해외 전원 환자에서 시작된 의료기관 내 전파 사례가 확인되었다[5,6]. 이에 따라 의료기관을 중심으로 한 전파 가능성과 지속적인 발생에 대비한 국가 감시ㆍ관리체계 마련의 필요성이 제기되었다. 국내 발생 현황과 의료기관 감염관리 실태를 분석하고[6,7], 전문가 자문 및 관련 위원회 심의를 거쳐 칸디다 오리스 감염증을 제4급 법정감염병 및 의료관련감염병으로 지정하였다. 칸디다 오리스는 아직 국내 발생 규모가 제한적이고 역학적 특성이 충분히 규명되지 않은 단계로, 국내 발생 현황을 신속하게 파악하면서 기존 의료관련감염병 표본감시체계를 즉시 활용할 수 있는 감시체계가 필요하였다. 이에 따라 전국 의료관련감염병 표본감시기관을 기반으로 한 제4급 법정감염병 관리체계를 우선 구축하고, 축적되는 역학자료를 바탕으로 향후 감시수준과 신고기준을 지속적으로 보완하는 단계적 접근 전략을 채택하였다. 본 보고서는 칸디다 오리스 감염증의 제4급 법정감염병 지정 과정과 국가 감시ㆍ관리체계 구축 내용을 소개하고, 신고체계, 감염관리, 치료지원 및 보완적 감시체계 운영을 포함한 국가 관리전략을 제시하고자 한다.

## 방 법

### 1. 국내외 발생 현황 및 관리체계 검토

칸디다 오리스 감염증의 국가 관리체계 구축을 위하여 국내외 발생 현황과 관리 동향을 검토하였다. 국내에서는 칸디다 오리스 발생 사례, 국내 Clade I 발생 현황 및 의료기관 내 집단발생 사례를 검토하였으며, 의료기관의 감염관리 수준과 관리체계 운영 현황을 함께 분석하였다. 국외 자료는 WHO, CDC 및 ECDC에서 발간한 관리지침과 권고사항을 중심으로 검토하였으며, 주요 국가의 법정감염병 관리체계와 의료기관 감염관리 정책을 비교ㆍ분석하였다[2-4].

### 2. 국내 의료기관 실태조사

국내 의료기관의 칸디다 오리스 발생 현황과 감염관리 수준을 파악하기 위하여 2025년 전국 의료기관 155개소를 대상으로 실태조사를 실시하였다. 실태조사에서는 의료기관별 칸디다 오리스 발생 여부, 검사 수행 현황, 환자관리 및 격리조치, 환경관리, 감염예방 및 관리체계 운영 현황 등을 조사하였으며, 조사 결과를 국가 감시ㆍ관리체계 구축과 신고기준 마련을 위한 기초자료로 활용하였다[7].

### 3. 제4급 법정감염병 지정 및 신고기준 마련

국내외 발생 현황과 실태조사 결과를 바탕으로 칸디다 오리스 감염증의 제4급 법정감염병 지정 필요성과 신고대상ㆍ신고기준ㆍ신고시기 및 운영방안을 검토하였다. 2024년 12월부터 전문가 자문회의, 관련 학회 검토, 공청회, 의료관련감염 전문위원회 및 감염병관리위원회 심의를 순차적으로 실시하였다.

### 4. 국가 감시ㆍ관리체계 구축 방안 검토

국내 발생 규모와 역학적 특성이 충분히 축적되지 않은 상황을 고려하여 기존 의료관련감염병 표본감시체계를 활용한 신고체계의 적용 가능성을 검토하였다. 아울러 제도 시행에 필요한 관리지침, 치료 권고안, 담당자 교육 및 격리치료 지원방안을 마련하고자 하였다.

### 5. 보완적 감시 추진 방안 마련

표본감시에서 수집되는 역학자료의 한계를 보완하기 위하여 의료기관에서 분리된 균주의 항진균제 감수성검사 및 분자역학 분석을 연계하는 보완적 감시 추진 방안을 마련하고자 하였다.

## 결 과

### 1. 제4급 법정감염병 지정 및 신고기준 마련

국내 발생 현황, 국외 관리 동향 및 전문가 의견을 종합적으로 검토한 결과, 칸디다 오리스 감염증은 국내 역학자료를 신속하게 축적하면서 기존 의료관련감염병 표본감시체계를 즉시 활용할 수 있는 관리체계가 적합한 것으로 판단하였다. 이에 따라 「질병관리청장이 지정하는 감염병의 종류」 및 「감염병 신고를 위한 진단기준」을 개정하여 칸디다 오리스 감염증을 제4급 법정감염병과 의료관련감염병으로 지정하고 신고대상과 진단기준을 마련하였다. 2026년 3월 29일부터 전국 의료관련감염병 표본감시기관에서 진단된 사례를 진단 후 7일 이내 신고하도록 하였다(표 1).

### 2. 국가 감시체계 구축

칸디다 오리스 감염증은 기존 의료관련감염병 표본감시체계를 활용하여 운영하도록 하였으며, 전국 368개 의료관련감염병 표본감시기관이 참여하는 감시체계를 구축하였다. 표본감시를 통하여 의료기관에서 발생하는 칸디다 오리스 감염증의 발생 규모와 환자 특성, 의료기관 유형별 분포 및 지역별 발생 현황을 지속적으로 수집하도록 하였으며, 이를 기반으로 국내 발생 양상과 전파 특성을 체계적으로 분석할 수 있도록 하였다.

### 3. 감염관리 및 치료 지원체계 마련

법정감염병 지정과 함께 「2026년도 칸디다 오리스 관리지침」과 「칸디다 오리스 치료 권고안」을 마련하여 전국 의료기관에 배포하였다[8,9]. 관리지침에는 환자의 조기 발견과 신고, 격리, 접촉주의, 환경소독 등 의료기관 내 전파 예방을 위한 감염관리 원칙을 제시하였으며, 주요 권고사항을 표 2에 요약하였다[8]. 또한 환자의 격리치료에 따른 의료기관의 부담을 완화하고 권고되는 감염관리 조치의 이행을 지원하기 위하여 격리실 입원료에 대한 건강보험 급여를 적용하였다[10].

### 4. 담당자 교육 및 제도 시행 지원

새로운 신고체계의 안정적인 운영을 위하여 제도 시행 이전 질병대응센터, 지방자치단체 및 전국 의료관련감염병 표본감시기관 담당자를 대상으로 신고기준과 신고절차, 감염관리 원칙 및 역학조사 방법에 대한 교육을 실시하였다. 이를 통해 법정감염병 지정에 따른 제도 변경사항을 공유하고, 기관 간 신고체계와 감염관리 절차를 표준화하여 제도의 안정적인 정착을 지원하였다.

### 5. 보완적 감시체계 구축

표본감시에서 수집되는 역학자료를 보완하기 위하여 중소ㆍ요양병원에서 분리된 칸디다 오리스 균주를 수집하고 항진균제 감수성검사와 분자역학 분석을 수행하는 보완적 감시체계를 구축하였다. 이를 통해 국내 유행 Clade 분포와 항진균제 내성 양상을 지속적으로 분석하고, 표본감시의 임상ㆍ역학정보와 연계하여 발생 특성과 전파 양상을 정밀하게 분석할 기반을 마련하였다. 이상의 국가 관리체계는 표본감시기관의 의무 신고를 중심으로 감염관리, 치료지원 및 보완적 감시가 연계되도록 구성하였다(표 3).

## 논 의

칸디다 오리스는 항진균제 내성, 의료기관 내 지속적인 전파 및 집단발생 가능성으로 인해 국제적으로 공중보건학적 중요성이 지속적으로 증가하고 있는 신종 진균 병원체이다. WHO는 2022년 Fungal Priority Pathogens List에서 칸디다 오리스를 최우선 대응군으로 지정하였으며[2], 미국과 유럽을 비롯한 여러 국가에서도 의료기관 중심의 감시체계와 감염관리 전략을 강화하고 있다[3,4]. 국내에서도 2018년 이후 Clade I 발생[5]과 의료기관 내 집단발생 및 전파 사례[5,6]가 확인되면서 국가 차원의 체계적인 감시와 관리 필요성이 제기되었다.

그러나 국내 발생 규모와 역학적 특성이 충분히 축적되지 않은 상황에서 즉시 전수감시를 시행하기에는 근거가 제한적이었다. 이에 따라 기존 의료관련감염병 표본감시체계를 활용하여 신속하게 국내 역학자료를 확보할 수 있는 제4급 법정감염병 관리체계를 우선 구축하였다. 이번 제도는 단순히 법정감염병을 지정하는 데 그치지 않고 신고체계, 감염관리, 치료지원 및 보완적 감시체계를 포함한 국가 관리체계를 마련하였다는 점에서 의의가 있다.

특히 기존 의료관련감염병 표본감시체계를 활용함으로써 새로운 감시체계 구축에 필요한 시간과 행정적 부담을 최소화하면서 전국 단위의 감시를 신속하게 시작할 수 있었다. 또한 관리지침과 치료 권고안을 마련하고[8,9] 격리실 입원료에 건강보험 급여를 적용함으로써 의료기관이 권고되는 감염관리 조치를 보다 적극적으로 시행할 수 있는 기반을 마련하였다[10]. 이는 의료기관의 경제적 부담을 완화하는 동시에 환자 격리와 의료기관 내 전파 차단을 지원하는 중요한 정책적 기반이 될 것으로 기대된다.

한편, 표본감시는 전국적인 발생 동향을 파악하는 데 효과적인 방법이지만 모든 발생 사례를 반영하기에는 한계가 있다. 이에 따라 보완적 감시를 통해 중소ㆍ요양병원에서 분리된 균주의 항진균제 감수성검사와 분자역학 분석을 수행하고, 임상 및 역학정보와 연계하여 국내 유행 Clade 분포와 전파 특성을 지속적으로 분석할 계획이다. 이러한 자료는 향후 국내 유행 양상을 보다 정확하게 파악하고 감시체계의 적절성과 신고기준을 재평가하는 근거로 활용될 수 있을 것이다. 결론적으로 칸디다 오리스 감염증의 제4급 법정감염병 지정은 국내 역학자료를 신속하게 축적하면서 의료기관 내 전파를 예방하기 위한 국가 관리체계를 마련한 첫 단계이다. 향후 표본감시와 보완적 감시를 연계하여 국내 역학정보를 지속적으로 축적하고 항진균제 감수성검사와 분자역학 정보를 활용함으로써 국내 상황에 적합한 감시ㆍ관리체계를 지속적으로 발전시켜 나갈 예정이다.

## Figures and Tables

**표 1:** 칸디다 오리스 감염증 신고기준

구분	신고기준
신고범위	환자, 병원체보유자
환자	혈액에서 칸디다 오리스가 분리된 사람
병원체보유자	혈액을 제외한 임상검체(단, 귀 검체 제외)에서 칸디다 오리스가 분리된 사람
신고주기	표본감시기관에서 진단 후 7일 이내 신고
임상증상	패혈증, 연조직 감염, 수술부위 감염과 같은 전신 감염 등의 증상 항진균제 치료에 반응하지 않는 지속적 감염증상
진단을 위한 검사기준	검체(귀 검체를 제외한 혈액, 소변 등)에서 칸디다 오리스 분리 동정

**표 2:** 칸디다 오리스의 의료기관 감염관리 핵심 권고사항

구분	핵심 권고사항
접촉주의	표준주의와 접촉주의를 적용한다. 환자ㆍ병원체 보유자 또는 주변 환경 접촉 전ㆍ후 손위생을 수행하고, 접촉 시 장갑과 가운을 착용한다.
환자 격리	가능한 한 1인실에 격리한다. 불가능한 경우 동일 병원균 감염ㆍ보균자끼리 코호트 격리하거나 병상 간 1 m 이상 거리를 유지하고 물리적 차단막을 설치한다. 입원기간 동안 지속 격리를 권고한다.
의료기구ㆍ환경관리	의료기구는 환자 전용 또는 일회용으로 사용한다. 공용기구와 고접촉 환경표면을 철저히 세척ㆍ소독하고, 환자 퇴실 후 종말소독을 실시한다.
선별검사ㆍ접촉자 관리	감염ㆍ보균 과거력이 있거나 역학적 연관성이 있는 환자에게 선별검사를 고려한다. 하나의 면봉으로 양쪽 겨드랑이와 양쪽 서혜부를 통합 채취한다.
이동ㆍ전원 관리	불필요한 환자 이동을 제한하고, 이동ㆍ전원 시 감염ㆍ보균 상태와 적용 중인 감염관리 조치를 전달한다.

**표 3:** 칸디다 오리스 감염증 국가 감시ㆍ관리체계

신고체계	감염관리	치료지원	보완적 감시
368개 표본감시기관 진단 후 7일 이내 신고	조기 발견ㆍ격리, 접촉주의ㆍ 환경소독	치료 권고안, 격리실 입원료 급여	균주 수집, 항진균제 감수성ㆍ 분자역학 분석
